# Bias in Environmental Cohort Studies: The Example of Bone Lead and Mortality

**DOI:** 10.1289/ehp.123-A288

**Published:** 2015-11-01

**Authors:** Carol Potera

**Affiliations:** Carol Potera, based in Montana, also writes for *Microbe*, *Genetic Engineering New*s, and the *American Journal of Nursing*.

Unrecognized biases in prospective environmental cohort studies may result in under- or overestimating the health effects of the exposure under investigation. In this issue of *EHP*, researchers examine the problem of bias using data on lead exposure and mortality in men and directed acyclic graphs (DAGs) to illustrate causal relationships between variables that could bias results.^1^

The study data came from 835 white male participants, average age 67 years, who were part of the Normative Aging Study (NAS), which began in 1963. Between 1991 and 1999, the men had undergone measurement of lead in their patellas. For the current analysis, the researchers looked at associations between patella lead and mortality from all causes, from cardiovascular disease, and from ischemic heart disease. Bone lead, rather than blood lead, is a better biomarker for cumulative environmental exposure,^2^ and patella lead in particular has been associated in past studies with risk of ischemic heart disease death.^3^

**Figure:**
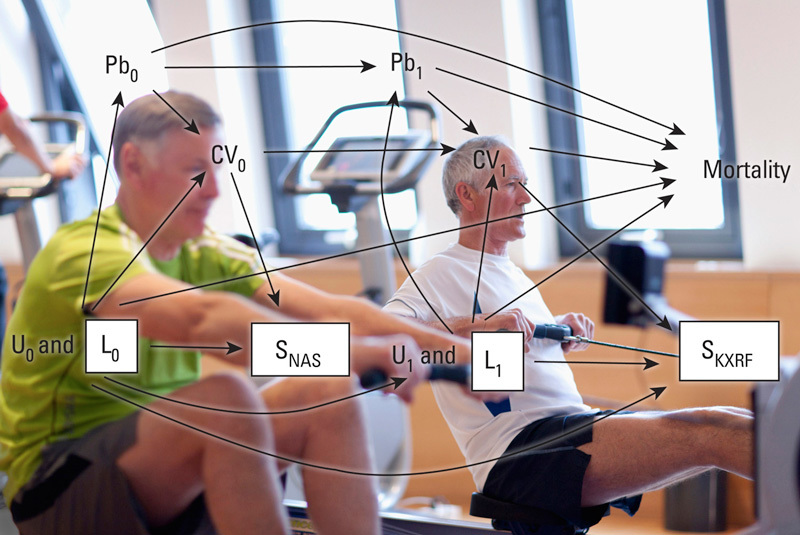
Directed acyclic graphs helped researchers pinpoint areas where bias related to recruitment and participation may have affected the Normative Aging Study and similar studies. © Echo/Getty Images; Weisskopf et al. (2015)^1^

The authors used DAGs to visualize relationships among variables that applied to the NAS participants. DAGs link variables with directional arrows to identify those that may bias results. The present study focused on potential errors related to participant selection and participation issues that may affect other cohort studies used in environmental health research. By explicitly visualizing assumptions with DAGs, researchers can identify where bias creeps in and find ways to avoid it.^4^ “Researchers don’t always recognize the assumptions they make when analyzing data,” says study leader Marc Weisskopf, an epidemiologist at the Harvard T.H. Chan School of Public Health.

As the team analyzed the DAGs, they realized that age at enrollment appeared to be influencing the risk associations. Overall, men with the highest patella lead levels were twice as likely as those with the lowest lead levels to die from ischemic heart disease. However, among the subset of 637 men who were 45 or younger when they entered the NAS cohort, those with the highest patella lead levels were estimated to be 4.6 times as likely to die from ischemic heart disease as those with the lowest levels. When the data were further adjusted to account for men who dropped out of the cohort, the estimated risk among younger men with the highest lead levels climbed to 5.2 times that of younger men with the lowest exposures.^1^

Men who were older when they enrolled in the NAS likely did not represent older men in general. That’s because many older men have cardiovascular conditions, which makes them less likely to participate in studies.^5^ Furthermore, the NAS specifically did not accept participants with pre-existing cardiovascular disease.^1^

In contrast, because younger people tend to be healthier, their health status is less likely to influence whether they enter a study. Ideally, long-term studies such as the NAS would follow younger participants for a longer time to avoid recruitment biases. However, the longer a cohort is followed, the more expensive the study becomes.

“This is particularly problematic with environmental exposures that are related to health conditions that affect people’s likelihood of participating in studies,” Weisskopf says. “Younger people are healthier to start, and it’s less likely that a health condition drives who enters a study.”

Socioeconomic status presents another source of potential bias. Less affluent people are both more likely to be exposed to environmental toxicants and less likely to participate in epidemiological studies, and those who do enroll are more likely to drop out.^6^ “It’s not random who drops out of a cohort,” notes Weisskopf.

Aside from the bias-related findings, the results suggest that previous estimates of lead’s influence on mortality may have been overly conservative. “They hint that we may be underestimating the health effects of other environmental toxicant exposures as well, especially in the context of health outcomes in older people and/or exposures associated with selection and attrition,” says Jennifer Weuve, an associate professor of internal medicine at Chicago’s Rush Medical College, who was not involved in the analysis. She says the methods used by Weisskopf’s team could be applied to other environmental epidemiology cohorts to improve estimates of health risks.

## References

[1] Weisskopf MG Biased exposure–health effect estimates from selection cohort studies: are environmental studies at particular risk? Environ Health Perspect 123 11 1113 1122 2015; 10.1289/ehp.1408888 25956004 PMC4629739

[2] Hu H Bone lead as a biological marker in epidemiological studies of chronic toxicity: conceptual paradigms. Environ Health Perspect 106 1 1 8 1998; PMID:9417769 10.1289/ehp.981061 PMC1532948

[3] Weisskopf MG A prospective study of bone lead concentration and death from all causes, cardiovascular diseases, and cancer in the Department of Veterans Affairs Normative Aging Study. Circulation 120 12 1056 1064 2009; 10.1161/CIRCULATIONAHA.108.827121 19738141 PMC2760410

[4] Greenland S Causal diagrams for epidemiological research. Epidemiology 10 1 37 48 1999; PMID:9888278

[5] Anstey KJ Luszcz MA Selective non-response to clinical assessment in the longitudinal study of aging: implications for estimating population levels of cognitive function and dementia. Int J Geriatr Psychiatry 17 8 704 709 2002; 10.1002/gps.651 12211118

[6] Howe LD Loss to follow-up in cohort studies: bias in estimates of socioeconomic inequalities. Epidemiology 24 1 1 9 2013; 10.1097/EDE.0b013e31827623b1 23211345 PMC5102324

