# Erratum: “Bias in Environmental Cohort Studies: The Example of Bone Lead and Mortality”

**DOI:** 10.1289/ehp.124-A10

**Published:** 2016-01-01

**Authors:** Carol Potera

Environ Health Perspect 123:A288 (2015); http://dx.doi.org/10.1289/ehp.123-A288

Jennifer Weuve was incorrectly identified as an assistant professor. Her correct title is associate professor.

*EHP* regrets the error.

